# Draft Genome of *Halomonas lionensis* RHS90^T^, a Stress-Tolerant Gammaproteobacterium Isolated from Mediterranean Sea Sediments

**DOI:** 10.1128/genomeA.00311-17

**Published:** 2017-06-08

**Authors:** Frédéric Gaboyer, Loïs Maignien, Mohamed Jebbar, Karine Alain

**Affiliations:** a Centre de Biophysique Moléculaire (CBM)–UPR4301, Centre National de la Recherche Scientifique, Orléans, France; b CNRS, IUEM–UMR 6197, Laboratoire de Microbiologie des Environnements Extrêmes (LM2E), Plouzané, France; c Université de Bretagne Occidentale (UBO, UBL), Institut Universitaire Européen de la Mer (IUEM)–UMR 6197, Laboratoire de Microbiologie des Environnements Extrêmes (LM2E), Plouzané, France; d Ifremer, UMR 6197, Laboratoire de Microbiologie des Environnements Extrêmes (LM2E), Plouzané, France

## Abstract

Members of the genus *Halomonas* are physiologically versatile and harbor ecological adaptations enabling the colonization of contrasted environments. We present here the draft genome of *Halomonas lionensis* RHS90^T^, isolated from Mediterranean Sea sediments. Numerous genes related to stress tolerance, DNA repair, or external signal-sensing systems were predicted, which could represent selective advantages of this marine bacterium.

## GENOME ANNOUNCEMENT

Members of Halophilic species belonging to the genus *Halomonas* (*Gammaproteobacteria*) are found in widespread habitats with varying environmental conditions. Cultural approaches first described the presence of *Halomonas* spp. in hypersaline environments (hypersaline lakes, solar salterns, or marine environments, including the salt-saturated Dead Sea) (1). Molecular tools further described the presence of *Halomonas* DNA sequences in many environments, saline or not, such as biofilms, murals, and human blood. Extreme environments are not spared by the genus *Halomonas*, as this taxon has been isolated from the deep sea, hydrothermal vents, subsurface sediments, and the oceanic crust (2).

Biogeography suggests that *Halomonas* spp. have physiological plasticity and adaptive features to cope with such different environmental conditions, making the genus *Halomonas* an appropriate model for studying ecological adaptations of generalists. Moreover, *Halomonas* spp. are also of industrial interest due to their ability to synthesize bioplastics (polyhydroxyalkanoates) and osmoprotectants (ectoine, glycine-betaine, and derivatives).

*Halomonas lionensis* RHS90^T^ is an aerobic heterotroph, isolated from Mediterranean Sea sediments, that synthesizes osmoprotectants under hypersaline conditions and stores polyhydroxyalkanoates (3). *H. lionensis* is also stress-tolerant and is able to cope with high concentrations of metals, high pressure, or starvation (3). In an effort to better understand the adaptive strategies that explain the ecological success of *Halomonas* spp., the genome of *H. lionensis* RHS90^T^ was sequenced.

Whole-genome shotgun sequencing was performed with Illumina MiSeq 2 × 250-bp-reads at 586× mean coverage (Fasteris society, Plan-les Ouattes, Switzerland). The genome was assembled with Velvet (4) using the GenOuest Galaxy platform (5) with a 34-bp *k*-mer, leading to 103 contigs >1,000 bp. The longest contig was 312,662 bp long, and the *N*_50 _was 111 246 bp. The open reading frames were detected with Glimmer version 3.0 and annotated with the RAST annotation service (6) and with the MicroScope Microbial Genome Annotation and Analysis Platform (MaGe) (7). Transfer RNAs were found with tRNAscan-SE (8).

The total assembly size was 3.90 Mb long with a G+C content of 55.9%. It encodes 4,734 putative genes and has a single 16S rRNA operon with a coding density of 0.77. A total of 78.4% and 52% of genes could be classified, respectively, in the Cluster of Orthologous Groups (COG) or SEED databases. We found many genes possibly explaining its stress tolerance, such as metallotolerance genes (72 predicted genes), oxidative stress genes (50 predicted genes), genes for carbon starvation response and cold shock response, or a complete DNA repair system (nucleotide excision repair, base excision repair, mismatch repair, SOS response or homologous recombination). Numerous histidine kinases and response regulators were predicted that could enable *H. lionensis* to sense external signals. The halophilic adaptation of *H. lionensis* RHS90^T^ was suggested by acidic-shifted pI values of its proteome. Genes for polyhydroxyalkanoate and osmoprotectant (ectoine, betaine, and polyol) synthesis and uptake were also predicted. Notably, the presence of two prophages, CRISPR regions, and genes for integrases and transposases points out a complex evolutionary history with genomic rearrangements. Based on 16S rRNA similarity, the closest sequenced *Halomonas* sp. is *Halomonas* sp. GFAJ1 (98% 16S rRNA identity) with 85.5% average nucleotide identity.

### Accession number(s).

This whole-genome shotgun project has been deposited at DDBJ/ENA/GenBank under the accession number MWVI00000000. The version described in this paper is the first version.
